# Schizophrenia spectrum participants have reduced visual contrast sensitivity to chromatic (red/green) and luminance (light/dark) stimuli: new insights into information processing, visual channel function, and antipsychotic effects

**DOI:** 10.3389/fpsyg.2013.00535

**Published:** 2013-08-20

**Authors:** Kristin S. Cadenhead, Karen Dobkins, Jessica McGovern, Kathleen Shafer

**Affiliations:** ^1^Department of Psychiatry, University of California San DiegoLa Jolla, CA, USA; ^2^Department of Psychology, University of California San DiegoLa Jolla, CA, USA

**Keywords:** schizophrenia, schizotypal, visual contrast sensitivity, magnocellular, parvocellular

## Abstract

**Background:** Individuals with schizophrenia spectrum diagnoses have deficient visual information processing as assessed by a variety of paradigms including visual backward masking, motion perception and visual contrast sensitivity (VCS). In the present study, the VCS paradigm was used to investigate potential differences in magnocellular (M) vs. parvocellular (P) channel function that might account for the observed information processing deficits of schizophrenia spectrum patients. Specifically, VCS for near threshold luminance (black/white) stimuli is known to be governed primarily by the M channel, while VCS for near threshold chromatic (red/green) stimuli is governed by the P channel.

**Methods:** VCS for luminance and chromatic stimuli (counterphase-reversing sinusoidal gratings, 1.22 c/degree, 8.3 Hz) was assessed in 53 patients with schizophrenia (including 5 off antipsychotic medication), 22 individuals diagnosed with schizotypal personality disorder and 53 healthy comparison subjects.

**Results:** Schizophrenia spectrum groups demonstrated reduced VCS in both conditions relative to normals, and there was no significant group by condition interaction effect. *Post-hoc* analyses suggest that it was the patients with schizophrenia on antipsychotic medication as well as SPD participants who accounted for the deficits in the luminance condition.

**Conclusions:** These results demonstrate visual information processing deficits in schizophrenia spectrum populations but do not support the notion of selective abnormalities in the function of subcortical channels as suggested by previous studies. Further work is needed in a longitudinal design to further assess VCS as a vulnerability marker for psychosis as well as the effect of antipsychotic agents on performance in schizophrenia spectrum populations.

## Introduction

Patients with schizophrenia (SZ), their first degree relatives and individuals diagnosed with schizotypal personality disorder (SPD) have deficient visual information processing as assessed by a variety of paradigms including visual backward masking (VBM), motion perception, spatial localization, eye tracking and visual contrast sensitivity (VCS) (Braff and Saccuzzo, 1981; Schwartz and Winstead, 1985; Schwartz et al., 1987, 1988; Green et al., 1994; O'Donnell et al., 1996; Saccuzzo et al., 1996; Cadenhead et al., 1998; Slaghuis, 1998; Chen et al., 1999; Green and Nuechterlein, 1999; Slaghuis and Curran, 1999; Keri et al., 2000, 2002; Butler et al., 2001). With increasing knowledge of information processing in the visual system, it has been possible to apply newer paradigms in SZ spectrum populations in order to specify the underlying mechanisms and possible neural substrates responsible for the observed deficits.

Physiological and anatomical data have documented the existence of two major subcortical pathways that originate in the eye and project to primary visual cortex, the magnocellular (M) and parvocellular (P) pathways (Breitmeyer and Ganz, 1976; Lennie, 1980; Kaplan and Shapley, 1986; Livingstone and Hubel, 1987; Bassi and Lehmkuhle, 1990). The M pathway is sensitive to low spatial frequency patterns, high temporal frequencies, and exhibits transient (on/off) responses to visual stimuli (Breitmeyer and Ganz, 1976; Lennie, 1980; Kaplan and Shapley, 1986; Livingstone and Hubel, 1987; Bassi and Lehmkuhle, 1990). By contrast, the P pathway is sensitive to high spatial frequency patterns, low temporal frequencies, and shows sustained responses to stimuli (Breitmeyer and Ganz, 1976; Lennie, 1980; Kaplan and Shapley, 1986; Livingstone and Hubel, 1987; Bassi and Lehmkuhle, 1990). Most relevant to the current study, the M pathway exhibits high contrast sensitivity to luminance (light/dark) patterns, and low contrast sensitivity to chromatic (red/green) patterns; conversely, the P pathway exhibits high contrast sensitivity to chromatic (red/green) patterns and low contrast sensitivity to luminance (light/dark) patterns (Lee et al., 1990; Shapley, 1990; Smith et al., 1995; Dobkins et al., 2009). In terms of cortical projections, the M pathway provides input to both the dorsal processing stream (the “where” system, which processes motion and space information) and the ventral processing stream (the “what” system, which processes object information) (Maunsell et al., 1990; Thiele et al., 2001), while the P pathway provides input primarily to the ventral processing stream.

To investigate the integrity of the M and P pathways in SZ, various psychophysical and neurophysiological approaches have been used that tap into the known visual properties of the two, or tap into the dorsal/ventral pathways that receive asymmetrical input from the M and P subcortical pathways (see above). In an early visual information processing study in SZ patients, Schwartz and Winstead (1982) assessed visible persistence by sequentially presenting sine-wave gratings of varying spatial frequencies, in order to differentially stimulate the parallel visual pathways. Both acute and chronic patients with SZ required longer inter-stimulus intervals (ISI's) than controls, with chronic patients performing most poorly when exposed to low spatial frequency stimuli that were utilized to bias activity in the magnocellular pathway. These findings were interpreted as either abnormal visual pathway (mainly M pathway) activity or a dysfunction in inhibitory mechanisms between the pathways that prevented the termination of visible persistence (Schwartz and Winstead, 1982).

Visual Backward Masking (VBM) paradigms have been developed that use different spatial frequency masks or require participants to locate (dorsal stream function) or identify (ventral stream function) target stimuli (Balogh and Merritt, 1987; Green et al., 1994; Cadenhead et al., 1998; Slaghuis and Curran, 1999). In the VBM paradigm, patients with SZ have been shown to exhibit deficits in the location, but not the identification, task when compared to normal comparison participants (Green et al., 1994; Cadenhead et al., 1998). Because locating targets in space is thought to be a dorsal stream function, and because the dorsal stream receives primarily M pathway input, this result has been interpreted as evidence of abnormal M pathway (subcortical) or dorsal stream (cortical) processing in SZ. As noted, there is considerable overlap between the M and P visual channels as early as the primary visual cortex (V1) and this interaction extends into the dorsal and ventral streams occurring thereafter (Kovacs et al., 1995; Sawatari and Callaway, 1996; Keri et al., 2000; Butler et al., 2007; Skottun and Skoyles, 2007) making it difficult to conclude from the VBM paradigm alone whether there are specific subcortical pathway deficits.

Perhaps one of the most straightforward ways in which M vs. P pathway processing in SZ has been investigated has been to measure VCS to stimuli designed to activate one pathway more than the other (Schwartz and Winstead, 1985, 1997; Schwartz et al., 1987, 1988; Slaghuis, 1998; Chen et al., 1999; Keri et al., 2000, 2002; Butler et al., 2001, 2009; Kiss et al., 2010; Kent et al., 2011; Halasz et al., 2013). VCS is defined as the inverse of the contrast needed in a stimulus (typically, a sinusoidal grating) in order for that stimulus to be just barely detectable, referred to as “contrast threshold.” The advantage of using the VCS paradigm is that it can *isolate* activity in a given pathway. The logic behind this approach is based on the fact that most stimuli will stimulate neurons in both the M and P pathways, for example, a high temporal frequency stimulus will activate both M and P neurons. However, given that one pathway is more sensitive to that stimulus (e.g., M neurons are more sensitive to high temporal frequency than are P neurons), one can assume that—*at contrast threshold*, only the more sensitive pathway (in this example, the M pathway) is responding to the stimulus (Smith and Edgar, 1991; Dobkins and Albright, 1995).

Using this approach, previous studies have measured VCS to different spatial/temporal frequencies, designed to isolate the M vs. P pathways. Slaghuis (1998; Slaghuis and Curran, 1999) examined VCS for stationary and moving stimuli across a range of spatial frequencies and reported that SZ patients with predominantly positive symptoms exhibited deficits at medium and high spatial frequencies, suggesting P pathway dysfunction, while the patients with predominantly negative symptoms performed poorly at all spatial frequencies, suggesting both M and P pathway dysfunction. Kéri and colleagues (2002) showed that patients with SZ have reduced VCS in the medium to high spatial frequency range in a stationary condition and over the full spatial frequency range in a moving condition. While the VCS dysfunction did not appear to be specific to either the M or P channel in the Kéri et al. study, more severe VCS deficits were associated with antipsychotic dose and Simpson Angus ratings of extrapyramidal side effects. Chen and colleagues (1999, 2003) did not find VCS deficits in SZ patients tested at low spatial frequency and varying temporal frequencies but reported that unmedicated patients (*N* = 6) exhibited VCS that was *greater* than that of normal participants. Furthermore, those patients on atypical antipsychotics were unimpaired while patients on typical antipsychotics had lower VCS. More recently, Kiss et al. (2010) have also reported that never medicated first episode patients with SZ (*N* = 20) have *greater* VCS in a pedestal condition that emphasizes M channel activity. The finding of reduced VCS in patients with SZ on antipsychotics in the Kéri and Chen et al. studies is similar to observations of reduced VCS in patients with Parkinson's Disease, suggesting that some of the effect may be secondary to modulation of spatiotemporal VCS functions by a hypodopaminergic state (Bulens et al., 1986, 1987, 1989; Bodis-Wollner et al., 1987). O'Donnell et al. (2006), however, found that both medicated and unmedicated patients with SZ were impaired relative to normals across both high and low spatial frequencies. In the same study, individuals meeting the DSM-IV criteria for SPD did not differ from normals. Similarly, Kent et al. (2011) reported that SPD patients had VCS deficits at low temporal frequencies. Using a visual evoked potential paradigm, Butler and colleagues (Butler et al., 2001, 2009) examined near threshold luminance and chromatic contrast (to emphasize M or P visual pathway activity, respectively) in patients with SZ in two studies who showed lower response levels to stimuli that were M biased (low luminance contrast with large squares) while P biased stimuli (low chromatic contrast) did not differentiate them from normals. All but 2 of the participants in the Butler et al. study were receiving antipsychotic medications at the time of testing; therefore, a medication effect could not be excluded.

In summary, it is not clear from the current literature whether the observed visual information processing deficits in SZ spectrum patients are specific to M vs. P visual channel dysfunction. An important finding that has emerged is that dopamine modulation via D_2_ receptor blockade likely affects VCS in SZ patients and may account for the observed deficits across a range of paradigms. In the current study, we likewise capitalized on M and P pathway differences in luminance and chromatic contrast sensitivities, respectively. To this end, VCS was measured in patients with SZ and individuals meeting criteria for SPD. The SPD participants are important because they do not have many of the confounding variables such as acute psychosis or medication effects seen in a chronic SZ population yet represent a SZ spectrum phenotype. It was predicted that patients diagnosed with SZ and individuals meeting the criteria for SPD would have lower contrast sensitivities consistent with other studies but we wanted to determine whether these differences were specific to either visual channel. Finally, to better understand the relationship of antipsychotic medication to VCS, the effect of antipsychotic medication were examined separately in *post-hoc* analyses.

## Materials and methods

### Participants

Participants included 53 (40M/13F) patients with SZ, 22 (11M/11F) individuals who met criteria for SPD and 53 (30M/23F) healthy comparison (HC) subjects. Participants provided written informed consent after receiving an explanation of the study. Individuals with a history of major medical or neurological disorders or significant drug abuse in the past were excluded. Additionally, all participants were screened for current drug use using urine toxicology tests. HC subjects were recruited through newspaper advertisements and had no history of Axis I or II disorders as assessed by the Structured Clinical Interview for DSM-IV Disorders (SCID I and SCID II) nor any family history of SZ in a first or second degree relative by self report. Established inter-rater reliability for the SCID in our laboratory is 0.98 (Perry and Braff, 1998).

SZ patients were recruited through inpatient and outpatient facilities at UCSD, a long term care facility, and the San Diego Alliance for the Mentally Ill. All participants received the SCID to confirm the diagnosis of SZ. Five SZ patients were not receiving antipsychotic agents.

SPD participants were recruited from outpatient facilities at UCSD and by newspaper advertisements per our established methods (Cadenhead et al., 2000). Additional SPD participants were identified through screening of potential normal comparison participants. All SPD participants were assessed with the SCID I and the Structured Interview for DSM-IV Personality Disorders (SIDP) by one of the investigators (KSC) to identify the diagnosis of SPD. Two SPD participants were receiving a low dose typical antipsychotic (perphenazine 6 Mg qd or trifluoperazine 2 mg).

All participants were screened for color blindness using Ishihara's tests for color deficiency (Ishihara, 1986) and were reported to have corrected visual acuity of 20/50 or better as measured by the Snellen Eye Chart.

### Visual contrast sensitivity

#### Apparatus and stimuli

Stimuli were generated using a Power Macintosh 8100/80 PC computer and Nanao T2-17TS FlexScan Color Monitor (1152 × 870 pixels, 75 Hz). The 8-bit video board allowed for 256 discrete levels of luminance. The CIE coordinates for the monitor primaries were: Red (0.61, 0.342), Green (0.298, 0.588), and Blue (0.151, 0.064). The maximum output for the monitor was calibrated to equal energy white (CIE chromaticity coordinates = 0. 333, 0.333), and the voltage/luminance relationship was linearized independently for each of the three guns in the display, using a PR-650 Colorimeter (Photoresearch). The PR-650 was used for photometric measurements to standardize to V_λ_ isoluminance, as well as for spectroradiometric measurements to compute L and M cone modulations produced by our visual stimuli.

Stimuli were 1.22 cycles/degree horizontally-oriented sinusoidal gratings moving at a temporal frequency 8.33 Hz and are either luminance (light/dark) or chromatic (red/green). Each trial consisted of a luminance or chromatic stimulus, subtending a visual angle of 1.64 degrees (a total of 2 cycles), centered 2.28 degree to the left or right of a fixation cross in screen center, for a duration of 120 Ms. Motion was produced by phase-shifting sinusoidal gratings at regular intervals in sync with the vertical refresh of the video monitor (75 Hz).

***Luminance (light/dark) gratings***. Luminance-defined gratings were produced by sinusoidally modulating the red and green phosphors in phase (with a small amount of blue gun also added in phase to match the mean chromaticity of the chromatic gratings). For luminance stimuli, r.m.s. (root mean square) cone contrast values directly correspond to the conventional Michelson contrast: [(L_max_ − L_min_)/(L_max_ + L_min_)], and cone contrasts up to 100% are readily produced. Although the calibration techniques allow for a specified amount of contrast, the actual luminance contrast of all stimuli was verified using the PR-650 Colorimeter.

***Chromatic (red/green) gratings***. Chromatic red/green gratings were produced by sinusoidally modulating the red and green phosphors 180 degrees out of phase, with a small amount of blue primary added in phase with the red portion of the grating so as to prevent modulation of the short-wavelength-sensitive (S) cones (Dobkins and Teller, 1996). The cone contrast of these stimuli was determined by spectroradiometry (with the PR-650) as described in detail previously (Gunther and Dobkins, 2002).

The red/green isoluminance point for each participant was determined using a 20 trial task during which participants were asked to adjust (increase or decrease) the luminance contrast (interval step = 0.5%) in a moving red/green grating (r.m.s. cone contrast = 7.2%) to the point where the pattern was no longer salient (not visible, faded, jagged or blurry). The mean isoluminance point was used in the chromatic portion of the contrast sensitivity paradigm (below).

#### Psychophysical paradigm

The VCS paradigm consisted of a standard 2-alternative-forced-choice task with feedback. Participants sat in a comfortable chair and rest their head in a chin rest. They were asked to fixate on a small “+” symbol in the center of a monitor 57 cm away. The participant started each trial with a key press on the standard computer keyboard, after which a grating stimulus (luminance or chromatic) of varying contrast appeared on the left or right side of the display. After the stimulus disappeared, the participant reported its location (left or right) using color-coded keys. Using a method of constant stimuli paradigm, six different levels of contrast were presented for each condition, presented randomly across trials. Participants were given 100 practice trials to familiarize them with the task. The experimental session contained 360 trials (180 trials for each the luminance and chromatic conditions) and lasted approximately 45 Min. The experiment was self-paced and participants were encouraged to take breaks as often as needed.

#### Data analysis

***Contrast sensitivities***. Psychometric curves were fit to data using Weibull functions and maximum likelihood analysis (Weibull, 1958; Watson, 1979). Contrast threshold was defined as the contrast yielding 75% correct performance. In extreme cases where the performance of a subject was poor in both the luminance and chromatic conditions so that no threshold could be determined from the Weibull, the subject was excluded (1 SPD and 7 SZ). All thresholds were analyzed in terms of r.m.s. cone contrast. Contrast sensitivity was determined from the inverse of threshold (i.e., sensitivity = 1/threshold). Raw contrast sensitivity data was log_10_ transformed, because log but not linear sensitivities conform to normal distributions. Data were analyzed in Two-Way ANOVAs that included the stimulus type (luminance and chromatic) and participant group.

## Results

### Demographics

Table 1 presents the demographic data of subjects included in the analysis. The groups did not differ in age [*F*_(2, 127)_ = 1.8, ns] or ratio of males to females (Pearson Chi Square Value = 5.3, ns) but education differed between the groups [*F*_(2, 119)_ = 22.4, *p* < 0.001] with SZ patients having significantly fewer years of completed education compared to SPD and HC participants (both *p* < 0.001). As expected, SZ patients had poorer functioning (GAF: *t* = 3.9, *p* < 0.001) and more symptoms (SANS: *t* = 4.9, *p* < 0.001; SAPS: *t* = 2.8, *p* < 0.001) relative to SPD subjects.

**Table 1:** **Demographics and visual contrast sensitivities**.

	**HC (*N* = 53)**	**SPD (*N* = 21)**	**SZ (*N* = 46)**
Age (SD)	33.8 (9.2)	37.8 (9.3)	35.7 (11.3)
Education (SD)	15.2 (2.2)	14.3 (2.6)	11.9 (2.7)
Gender (% male)	56.6	52.4	76.1
GAF (SD)		58.2 (14.9)	43.2 (14.1)
Global SAPS (SD)		6.0 (2.7)	8.8 (5.0)
Global SANS (SD)		4.7 (3.5)	10.9 (5.1)

*GAF, global assessment of functioning; SAPS, schedule for the assessment of positive symptoms; SANS, schedule for the assessment of negative symptoms*.

### Group mean contrast sensitivity

The results of a Two-Way ANOVA revealed a significant main effect of participant group [*F*_(2, 118)_ = 8.81, *p* < 0.001]. *Post-hoc* *t*-tests indicated that this effect was driven by significantly lower contrast sensitivity (collapsed across luminance and chromatic) in both SZ spectrum groups as compared to HC subjects (*p* < 0.05 vs. SPD and *p* < 0.001 vs. SZ). The SZ and SPD participants did not significantly differ from each other. There was no interaction between participant group and stimulus condition [*F*_(2, 117)_ = 0.56, ns], indicating that the group differences did not differ between luminance and chromatic stimuli. There were no significant gender or interaction effects with gender, and therefore groups were collapsed across gender for the analyses presented below. Visual contrast sensitivity in the luminance but not the chromatic condition was significantly correlated with age while neither condition was associated with symptoms or functioning measures above.

### Medication effects

**Figure 1:**
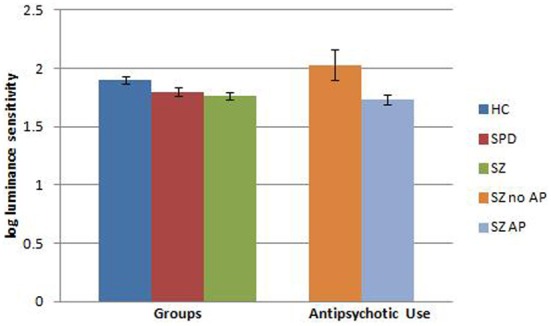
**Visual contrast sensitivity (error bars represent SEM) in the luminance condition comparing healthy comparison (HC), schizotypal personality disorder (SPD), and schizophrenia (SZ) subjects.** Data from unmedicated SZ subjects as well as SZ subjects on antipsychotic (AP) medication are also shown relative to the original group analysis.

**Figure 2:**
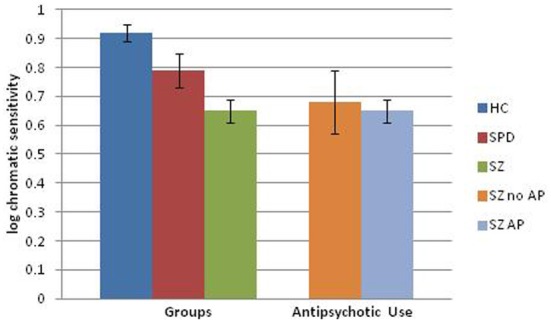
**Visual contrast sensitivity (error bars represent SEM) in the chromatic condition comparing healthy comparison (HC), schizotypal personality disorder (SPD), and schizophrenia (SZ) subjects.** Data from unmedicated SZ subjects as well as SZ subjects on an antipsychotic (AP) medication are also shown relative to the original group analysis.

Because of the potential effects of antipsychotic agents on visual information processing, we conducted further analyses in which the 2 SPD participants on antipsychotic medication were removed from the analysis and the 41 patients with SZ on antipsychotics (SZ+AP) were compared to 5 patients with SZ who were not on antipsychotics (SZ-AP), the 53 HC and 18 SPD subjects (see Figures 1, 2). A two-factor ANOVA revealed a significant effect of group [*F*_(3, 114)_ = 6.77, *p* < 0.001], driven by HC vs. SZ patients on antipsychotics (*p* < 0.001) and HC vs. SPD (*p* < 0.05), and a significant group by condition interaction [*F*_(3, 114)_ = 3.86, *p* < 0.01]. To investigate this interaction further, we performed separate one-factor ANOVAs for the luminance and chromatic conditions. Both analyses showed a significant main effect of group [luminance: *F*_(3, 117)_ = 7.56, *p* < 0.001; chromatic: *F*_(3, 117)_ = 5.03, *p* < 0.005], but for different reasons. For the luminance condition, *post-hoc* *t*-tests revealed impaired luminance contrast sensitivity in the SZ patients on antipsychotics as compared to HC (*p* < 0.001), yet *higher* luminance contrast sensitivity in unmedicated SZ patients vs. antipsychotic medicated patients with SZ (*p* < 0.005), and no significant difference between unmedicated SZ patients and HC subjects. Inspection of the data reveals that the unmedicated SZ patient have greater luminance contrast sensitivity than HCs (effect size = 0.62) but this analysis was not significant (*p* = 0.16). Power analyses indicate that at least 12 subjects per group would be needed to achieve a statistically significant result (power 0.80, *p* < 0.05). For the chromatic condition, *post-hoc* tests revealed impaired chromatic contrast sensitivity in antipsychotic treated SZ patients as well as unmedicated patients with SZ and SPD subjects, as compared to HC subjects (*p* < 0.001, *p* < 0.05, and *p* < 0.05, respectively) while the antipsychotic treated vs. unmedicated patients did not differ from each other. Follow-up correlations between chlorpromazine equivalents and contrast sensitivities within the antipsychotic treated sample of patients were not significant. In summary, patients with SZ on antipsychotic medication have impaired VCS in both luminance and chromatic conditions and the small group of unmedicated patients had normal luminance contrast and impaired chromatic contrast. Or stated differently, if we could remove the effects of medication in SZ patients, they might as a group show atypically high luminance contrast sensitivity, yet impaired chromatic contrast sensitivity.

## Discussion

Schizophrenia spectrum groups demonstrated reduced VCS in both luminance and chromatic conditions relative to healthy subjects, but the relative sensitivity to luminance vs. chromatic stimuli did not differ between groups. These results replicate previous findings of visual information processing deficits in SZ spectrum populations but do not support the notion of selective abnormalities in the function of the subcortical M pathway as suggested by previous studies (Butler et al., 2007). Instead, the results suggest either abnormalities in both M and P pathways or a more general visual processing deficit at some point further downstream in individuals diagnosed with SZ spectrum illness. The M and P pathways are responsible for relaying specific visual information from the retina, through the lateral geniculate nucleus of the thalamus, to primary visual cortex (V1), and elsewhere in the cortex. A contrast detection deficit may arise from dysfunction at any of these levels.

As noted by previous investigators (Bodis-Wollner et al., 1982; Bulens et al., 1986, 1987, 1989; Keri et al., 2002; Chen et al., 2003), the possibility of medication effects accounting for the visual information processing deficits cannot be entirely ruled out. The differences between the SZ patients and the HC subjects in the luminance VCS condition were no longer present when the small sample of patients (*N* = 5) not receiving antipsychotics were compared to HCs. In fact, in the luminance condition, the five unmedicated SZ patients had contrast sensitivities *greater than* those of the antipsychotic treated patients (*p* < 0.005) and HC (*p* = 0.16, *d* = 0.62) subjects, consistent with the findings by Chen and colleagues (2003) in six unmedicated SZ patients and the twenty unmedicated SZ patients in the Kiss et al. study (2010). It is possible that the hypodopaminergic effect of the antipsychotics accounted for the original difference between groups in the luminance condition while a hyperdopaminergic state may increase contrast sensitivity as observed in the unmedicated patients with SZ. Interpretation of the current findings in unmedicated patients, are limited by the small sample size but add to a growing literature that suggests evidence of overactive M channel activity.

It is known that dopamine neurotransmission (specifically, of the horizontal, amacrine, and interplexiform cells) in the retina is involved in regulating the strength of lateral inhibition and center-surround antagonism (Tagliati et al., 1994; Sannita, 1995; Djamgoz et al., 1997). A hyperdopaminergic state may enhance the center-surround processing which could in turn, enhance contrast sensitivity. Supporting the role of dopamine-modulation effects, Bodis-Wollner and colleagues (1982) found that Parkinson's disease patients treated with L-dopa and unmedicated SZ patients had faster visual evoked potentials than Parkinson's patients without L-dopa and SZ patients treated with typical antipsychotics. Similarly, a study by Harris and colleagues (1990) showed that after a therapeutic injection of depot antipsychotic, unmedicated patients with SZ (*N* = 8) had enhanced sensitivity at low (0.5 c/degree), and reduced sensitivity at medium (2 c/degree) and high (8 c/degree) spatial frequencies.

In contrast to the findings in SZ patients, the SPD participants, who were not taking antipsychotic medications, had deficits in both luminance and chromatic conditions supporting the notion of general visual information processing deficits in SZ spectrum populations that are not accounted for by medication effects. These results differ from the findings of O'Donnell and colleagues (2006) who reported that individuals with SPD did not differ at any spatial or temporal frequency from healthy subjects and performed significantly better than individuals with SZ. The results may be due to methodological differences. O'Donnell and colleagues did not have a chromatic condition and had a larger age range. Consistent with our findings, Kent and colleagues (2011) did find deficits in SPD at all spatial frequencies but the deficits were more prominent in a pedestal condition that emphasizes magnocellular activity. Whether or not SPD subjects have visual contrast deficits, it remains possible that a hyperdopaminegic state of unmedicated psychotic patients may be enhancing contrast sensitivity while SPD patients, who are characterized by only mild subsyndromal psychotic-like symptoms, tend to have more prominent deficit symptoms that may reflect hypodopaminergia that is accounting for the VCS deficits (Siever and Davis, 2004).

Given the present findings in SPD, it remains possible that the visual information processing deficits as indexed by VCS could represent trait markers for SZ spectrum illness. The identification of trait markers for SZ has implications for genetic studies as well as early identification of individuals at risk for SZ (Cadenhead, 2002). If reliable trait markers can be utilized in conjunction with clinical risk factors in identification of individuals in the prodromal stage of SZ, it may be possible to intervene earlier and prevent many of the devastating effects of a first psychotic break. Further understanding of the underlying neuropathology in the developing brain of individuals in the early stages of SZ could shed insight into targets for early intervention.

Methodological differences may play a role in discrepancies across VCS studies. Different visual contrast studies vary in how sensitivity is measured (e.g, staircase method, method of constant stimulus), stimuli (random dots vs. sinusoidal gratings), how the stimuli are presented (side-by-side or sequentially) and which spatial and temporal frequencies are in the range for biasing processing toward the M vs. P pathways. Further, whereas the present study used luminance (light/dark) and chromatic (red/green) stimuli to isolate M and P pathway activity, other studies varied spatial and temporal frequencies.

Moreover, there is a large variance between studies among the SZ spectrum participants included. Studies differ in participants' ages (O'Donnell et al., 2006), whether participants are chronic vs. acute case (Keri and Benedek, 2007), how long they have been on or off antipsychotics (Chen et al., 2003), and if positive vs. negative symptoms are analyzed separately (Slaghuis and Thompson, 2003). Furthermore, growing research has shown that SZ spectrum patients may vary in their dopamine and other neurotransmitter levels at different points during their illness (Uchida and Mamo, 2009). With increasing age, there is also a decline in D_2_ receptor binding (Antonini and Leenders, 1993). All of these factors suggest that chronic SZ coupled with years of dopamine-blocking antipsychotics may contribute to a greater hypodopaminergic state.

Clearly, further work is needed using a longitudinal design to chart the course of visual information processing deficits both on and off antipsychotic medication over the course of SZ from prodrome to acute to chronic forms of the illness. The use of VCS paradigms in combination with functional measures of brain activity may help to determine the mechanism by which such deficits occur and lead to better treatments that target specific information processing deficits without causing further impairment.

### Conflict of interest statement

The authors declare that the research was conducted in the absence of any commercial or financial relationships that could be construed as a potential conflict of interest.
